# Understanding depression in type 2 diabetes: a biological approach in observational studies

**DOI:** 10.12688/f1000research.13898.1

**Published:** 2018-08-14

**Authors:** Thomas van Sloten, Miranda Schram

**Affiliations:** 1 Department of Internal Medicine, Maastricht University Medical Centre, Maastricht, Netherlands; 2 CARIM School for Cardiovascular Diseases, Maastricht University, Maastricht , Netherlands; 3 Departments of Epidemiology and Arterial Mechanics, INSERM U970 and Paris-Cardiovascular Research Center, Paris, France; 4 Heart and Vascular Centre, Maastricht University Medical Centre, Maastricht , Netherlands

**Keywords:** type 2 diabetes, depression, hyperglycaemia, (micro-)vascular dysfunction, low-grade inflammation

## Abstract

Depression is twice as common in type 2 diabetes as in the general population and is associated with adverse health outcomes. Growing evidence suggest that type 2 diabetes and depression share biological mechanisms. This brief commentary discusses current understanding of shared biological pathways, focussing on hyperglycaemia, (micro)vascular dysfunction, and low-grade inflammation. Although there is accumulating evidence that these pathways are involved in the link between type 2 diabetes and depression, direct evidence of their temporal associations is lacking because of a paucity of longitudinal studies that focus on the pathobiology of both type 2 diabetes and depression.

## Introduction

Type 2 diabetes and depression are common, complex conditions that carry a large disease burden. Moreover, diabetes and depression commonly co-occur; individuals with type 2 diabetes have a doubled risk for depression as compared with individuals without diabetes ^1, 2^. Furthermore, individuals with depression have a 1.5 higher risk of diabetes ^3^. The joint presence of diabetes and depression is burdensome and is frequently overlooked despite the strong, negative impact on the quality of life of those who are affected. In addition, depression in diabetes is associated with higher health-care costs ^4^ and loss of productivity and higher absence from work ^5^. But this is not the full picture of the negative consequences of depression in diabetes. In longitudinal studies, patients with both diabetes and depression were found to have a considerably higher risk of developing microvascular and macrovascular complications of diabetes, in addition to a 50% higher mortality risk, as compared with patients without depression ^6^. Finally, depression appears to be highly persistent or recurrent in type 2 diabetes (or both) ^7^, suggesting either that depression is overlooked and thus untreated in patients with diabetes or that depression is resistant to currently available treatment or both.

This raises the need to unravel the causes of the close relationship between diabetes and depression. Currently, however, the aetiology of depression in diabetes is poorly understood. One interpretation that received quite some attention is that psychological factors, such as the burden of life with a chronic disorder and particular events in the disease course of diabetes (development of complications and start of insulin treatment), predispose diabetes patients to depression. However, the bidirectional relationship between type 2 diabetes and depression suggests that shared biological mechanisms may also underlie the link between type 2 diabetes and depression. Such biological mechanisms are likely to parallel those events that increase the burden of disease in diabetes and thus their importance may be overlooked when focussing only on the burden-of-disease hypothesis. Yet only few studies have investigated such potential shared biological mechanisms. In this short commentary, we will discuss the role of three, partly entangled major pathophysiologic pathways, namely hyperglycaemia, (micro)vascular dysfunction, and inflammation, as potential mechanisms or common ground for the development of depression in type 2 diabetes.

## Hyperglycaemia and depression

Hyperglycaemia itself may be a compelling regulator for mood states. Both fluctuations in plasma glucose as well as prolonged hyperglycaemia, as reflected in high HbA1c levels, may be involved in the development of depression in type 2 diabetes. The brain is particularly vulnerable to fluctuations in plasma glucose levels because neurons do not possess an active glucose transporter. As a result, high plasma glucose levels will have a direct effect on intraneuronal glucose levels. On a cellular level, these high intracellular glucose levels can activate the polyol pathway, thereby inducing oxidative stress and the enhanced formation of advanced glycation end products (AGEs), and both may lead to neuronal damage, which may eventually lead to depression ^8– 10^. In addition, increasing evidence suggests that hyperglycaemia leads to increased levels of cortisol ^11^, which is known to be involved in the development of depression.

Despite the plausibility of these mechanisms, it remains unclear whether there is a temporal association between blood glucose levels and the development of depression. A recent meta-analysis of longitudinal data on this topic ^12^ showed no increased risk for depressive symptoms in individuals with prediabetes or newly developed diabetes, whereas there was an increased risk for individuals with clinically identified diabetes. However, the relatively small sample size and methods used to assess depression (that is, questionnaire but not a diagnostic interview) hinder a conclusive answer to the question of whether hyperglycaemia is causally related to depression. In addition, recent cross-sectional data have shown that AGEs, which form as a result of prolonged hyperglycaemia, are independently associated with major depressive disorder as assessed with a diagnostic interview; this argues in favour of a role for hyperglycaemia in the development of depression ^13^. In order to address the question on the temporality of the association between hyperglycaemia and depression, there is a need for well-designed longitudinal population-based studies as well as mechanistic studies. These studies should assess continuous levels of hyperglycaemia parameters and determine depression on the basis of gold-standard assessment (that is, by interview) as opposed to questionnaires on depressive symptoms.

## Diabetes, depression, and (micro)vascular dysfunction

The vascular depression hypothesis proposes that vascular damage in frontal and subcortical regions of the brain, which are involved in mood regulation, may lead to depression in later life ^14^. In agreement with this hypothesis, recent longitudinal studies, as meta-analysed by van Agtmaal *et al*. ^15^ and Rensma *et al*. ^16^, have indeed identified that various brain-scanning markers of cerebral small vessel disease (for example, white matter hyperintensities and lacunar infarctions) are associated with a higher risk of depressive symptoms. The vascular depression hypothesis may be especially relevant in type 2 diabetes because diabetes is strongly linked to cerebrovascular damage. Previous studies have reported that diabetes is associated with cortical infarctions ^17^ and markers of more subtle vascular brain damage, including white matter hyperintensities ^18^ and lacunes of presumed vascular origin ^19, 20^.

Further evidence linking diabetes and depression via vascular damage comes from aetiologic studies that investigated the pathways of microvascular dysfunction and arterial stiffening. These are considered crucial pathways early in the development of cerebrovascular damage ^21, 22^ and are known to be strongly related to diabetes as well ^23^. The aforementioned meta-analysis by van Agtmaal *et al*. ^15^ identified that various markers of microvascular dysfunction (for example, endothelial-derived blood biomarkers) were associated with depression ^15^. These meta-analytic data showed small to moderate effect sizes and odds ratios ranging from 1.09 to 1.96 per standard deviation. Moreover, several cross-sectional studies have shown that arterial stiffness is higher in individuals with depression as compared with those without ^24– 28^. In addition, arterial stiffening has been associated with more depressive symptoms and this association was explained, or mediated, by brain-scanning markers of cerebral small vessel disease within the Ages Gene/Environment Susceptibility (AGES)-Reykjavik Study ^29^. However, longitudinal evidence for an association between arterial stiffness and depression is missing.

Multiple studies thus show consistent neuroimaging and vascular correlates of both type 2 diabetes and depression separately. However, almost no studies have investigated the two disorders together. There is a paucity of both cross-sectional and longitudinal studies that have investigated both conditions and these pathophysiologic mechanisms in a sufficiently detailed manner. Such studies are needed to establish whether (micro)vascular damage indeed underlies the association between diabetes and depression.

## Low-grade inflammation, diabetes, and depression

Low-grade inflammation is considered a key mediator of many chronic conditions. Type 2 diabetes is well known to be accompanied by systemic low-grade inflammation ^30^, which is considered an important mechanism in the development of its cardiovascular complications ^31^. Although the evidence is less extensive for depression, low-grade inflammation is indeed associated with incident depression ^32– 34^ and thus may represent a common biological pathway in the development of both type 2 diabetes and depression. Moreover, a recent meta-analysis shows that treatment resistance to antidepressants is associated with persistently elevated low-grade inflammation ^35^. This may indicate that treatment-resistant depression has a different aetiology that is not captured by the most commonly used antidepressants (for example, selective serotonin reuptake inhibitors). Furthermore, previous trials suggest that anti-inflammatory therapy may be beneficial in depression ^36^. The inflammatory pathway is likely to involve endothelial dysfunction and oxidative stress, and markers of these processes have been related to depression as well ^37, 38^. However, studies that specifically focus on this topic in relation to depression and type 2 diabetes are scarce ^39^, and cross-sectional in nature, and therefore cannot support any conclusions about causality.

In summary, the available data may suggest that low-grade inflammation is a common ground of depression as well as cardiovascular disease in type 2 diabetes. However, there is a paucity of longitudinal data that can provide more robust evidence on this association. Despite this conclusion, the potential of low-grade inflammation as a prognostic tool for choosing the treatment modalities in individuals with both depression and type 2 diabetes may be large. Medication that targets low-grade inflammation may have beneficial effects on both depression and cardiovascular disease risk in type 2 diabetes.

## Future directions

For all three pathophysiologic pathways discussed in this short commentary—hyperglycaemia, (micro)vascular dysfunction, and low-grade inflammation—greater understanding is needed on the temporal association between depression and type 2 diabetes. The vast majority of available studies have a cross-sectional design, thereby limiting the possibility of assessing the temporal sequence of the observed associations. Future studies should focus on a more detailed assessment of hyperglycaemia and depression in order to elucidate whether any causal association exists with depression. Although consistent associations on (micro)vascular dysfunction and low-grade inflammation with both type 2 diabetes and depression have been reported separately, almost no studies have investigated the two disorders together. Longitudinal studies across the life course are needed to assess the role of these potential shared mechanisms that link type 2 diabetes and depression. In addition, the biological mechanisms discussed in this commentary may well interfere with psychological mechanisms, as, for instance, is proposed in recent studies that link metabolism and psychopathology via the hypothalamic–pituitary axis, as suggested by the stress model ^40^. Further study is needed to elucidate these interrelationships as well. The ultimate goal of improving our understanding of the underlying pathophysiology of depression and diabetes is to find new targets to successfully prevent and treat the co-occurrence of type 2 diabetes and depression.

## Abbreviations

AGE, advanced glycation end product.
